# *LINE-1* methylation in visceral adipose tissue of severely obese individuals is associated with metabolic syndrome status and related phenotypes

**DOI:** 10.1186/1868-7083-4-10

**Published:** 2012-07-02

**Authors:** Valérie Turcot, André Tchernof, Yves Deshaies, Louis Pérusse, Alexandre Bélisle, Simon Marceau, Simon Biron, Odette Lescelleur, Laurent Biertho, Marie-Claude Vohl

**Affiliations:** 1 Institute of Nutraceuticals and Functional Foods, 2440 Hochelaga Blvd, Québec City, G1V 0A6, Canada; 2 Molecular Endocrinology and Genomics, CHUL Research Center, 2705 Laurier Blvd, Québec City, G1V 4G2, Canada; 3 Department of Food Sciences and Nutrition, Université Laval, 2425 de l’Agriculture Av, Québec City, G1V 0A6, Canada; 4 Centre de Recherche de l’Institut Universitaire de Cardiologie et de Pneumologie de Québec, 2725 Sainte-Foy Rd, Québec City, G1V 4G5, Canada; 5 Department of Medicine, Université Laval, 1050 de la Médecine Av, Québec City, G1V 0A6, Canada; 6 Department of Kinesiology, Université Laval, 2300 de la Terrasse Street, Québec City, G1V 0A6, Canada; 7 Genotyping Platform Team, McGill University and Génome Québec Innovation Center, 740 Docteur-Penfield Av, Montréal, H3A 1A4, Canada; 8 Department of Surgery, Université Laval, 1050 de la Médecine Av, Québec City, G1V 0A6, Canada

**Keywords:** Blood pressure, Epigenetics, Fasting glucose, Global DNA methylation, LINE-1, Metabolic syndrome, Severe obesity, Visceral adipose tissue

## Abstract

**Background:**

Epigenetic mechanisms may be involved in the regulation of genes found to be differentially expressed in the visceral adipose tissue (VAT) of severely obese subjects with (MetS+) versus without (MetS-) metabolic syndrome (MetS). Long interspersed nuclear element 1 (*LINE-1*) elements DNA methylation levels (%meth) in blood, a marker of global DNA methylation, have recently been associated with fasting glucose, blood lipids, heart diseases and stroke.

**Aim:**

To test whether *LINE-1*%meth levels in VAT are associated with MetS phenotypes and whether they can predict MetS risk in severely obese individuals.

**Methods:**

DNA was extracted from VAT of 34 men (MetS-: *n* = 14, MetS+: *n* = 20) and 152 premenopausal women (MetS-: *n* = 84; MetS+: *n* = 68) undergoing biliopancreatic diversion for the treatment of obesity. *LINE-1*%meth levels were assessed by pyrosequencing of sodium bisulfite-treated DNA.

**Results:**

The mean *LINE-1*%meth in VAT was of 75.8% (SD = 3.0%). Multiple linear regression analyses revealed that *LINE-1*%meth was negatively associated with fasting glucose levels (β = -0.04; *P* = 0.03), diastolic blood pressure (β =  -0.65; *P* = 0.03) and MetS status (β = -0.04; *P* = 0.004) after adjustments for the effects of age, sex, waist circumference (except for MetS status) and smoking. While dividing subjects into quartiles based on their *LINE-1*%meth (Q1 to Q4: lower %meth to higher %meth levels), greater risk were observed in the first (Q1: odds ratio (OR) = 4.37, *P* = 0.004) and the second (Q2: OR = 4.76, *P* = 0.002) quartiles compared to Q4 (1.00) when adjusting for age, sex and smoking.

**Conclusions:**

These results suggest that lower global DNA methylation, assessed by *LINE-1* repetitive elements methylation analysis, would be associated with a greater risk for MetS in the presence of obesity.

## Background

Accumulation of fat preferentially in the abdominal cavity is frequently associated with clustering of metabolic and inflammatory alterations, often referred to as metabolic syndrome (MetS) [1,2]. However, there exists a large heterogeneity in the development of these metabolic complications among obese individuals [3,4], which may partly be explained by impairment of adipose tissue function [5,6]. Previous studies observed differential gene expression patterns in visceral adipose tissue (VAT) of ‘metabolically healthy’ vs ‘metabolically unhealthy’ obese individuals [7-9]. It is thus possible that some of these differentially expressed genes may be functionally related to VAT dysfunction and MetS-related phenotypes. Although polymorphisms within several genes have previously been associated with both gene expression variability and MetS components [5], epigenetic mechanisms may also contribute to this variability.

Several epidemiological and animal studies demonstrated that a detrimental environment during fetal and postnatal periods, such as maternal obesity and gestational diabetes, is associated with increased risk of obesity and metabolic complications later in life [10,11]. These long-term complications would partly be explained by impaired organogenesis and cellular energy metabolism [12], as it may be the case regarding adipose tissue development and function [12-14]. Thus, impairments of normal development will ultimately change gene expression patterns in key metabolic pathways involved in obesity and MetS, which may potentially be mediated through epigenetic mechanisms [15,16]. DNA methylation is the most widely studied epigenetic phenomenon in humans. While association analyses between CpG methylation levels of candidate genes and complex diseases are increasing [17], an emerging literature uses CpG methylation of repetitive DNA sequences in disease association analyses [18]. This is the case for long interspersed nuclear element 1 (*LINE-1*) elements, which is a marker of the global DNA methylation of the genome [19]. *LINE-1* elements are a family of transposon-derived sequence repeats dispersed in the genome [20]. These repetitive elements are normally heavily methylated and their hypomethylation is commonly associated with cancer development and progression [21,22].

Interestingly, recent epidemiological studies also identified some associations between *LINE-1* methylation variability and ischemic heart disease and stroke [23], as well as with MetS phenotypes, such as plasma fasting glucose [24] and plasma lipid levels [24,25].

In view of the recent observations regarding blood *LINE-1* methylation levels and MetS-related phenotypes, the aim of the present study was to test whether *LINE-1* methylation levels in VAT are associated with MetS phenotypes and whether they can predict MetS risk in severely obese individuals.

## Methods

### Patient selection

The study subjects were severely obese Caucasian men (MetS-: *n* = 14; MetS+: *n* = 20) and premenopausal women (MetS-: *n* = 84; MetS+: *n* = 68) undergoing a biliopancreatic diversion with sleeve gastrectomy to treat obesity [26] at the Institut Universitaire de Cardiologie et de Pneumologie de Québec (Québec City, Canada) from June 2000 to June 2010, and for whom DNA samples from VAT were available. The study subjects were not taking any medications to treat MetS features and were not affected by type 2 diabetes mellitus [27]. Body weight, height, waist circumference, resting systolic and diastolic blood pressure were measured using standardized procedures [28]. The day of surgery, fasting blood samples were drawn into EDTA-containing tubes and centrifuged for plasma lipid and glucose concentration measurements [28]. VAT from the greater omentum was sampled during the surgery, as previously reported [26,29]. The diagnosis of MetS was based on the International Diabetes Federation (IDF) definition [30]. MetS + subjects had to be centrally obese (waist circumference ≥88 cm for women and ≥102 cm for men; USA cut-off values from the Adult Treatment Panel III [31]) with ≥2 other MetS criteria among the following: fasting plasma glucose ≥5.6 mmol/l, high-density lipoprotein (HDL)-cholesterol <1.29 mmol/l for women and <1.03 mmol/l for men, triglycerides ≥1.7 mmol/l, systolic blood pressure (SBP) ≥130 mmHg or diastolic blood pressure (DBP) ≥85 mmHg. All subjects provided a written informed consent to participate in this study, which received the approval of Université Laval Ethics Committee.

### Analysis of *LINE-1* CpG methylation in VAT

DNA was extracted from VAT using the DNeasy Blood & Tissue kit (Qiagen, Mississauga, Ontario, Canada), as recommended by the manufacturer. DNA extracts were stored at -80°C until quantitative methylation analysis using the pyrosequencing technology from Qiagen [32], which was performed by the McGill University and Genome Québec Innovation Center Genotyping Platform team (Montréal, Canada). DNA (1 μg) was treated with sodium bisulfite followed by a purification step using the EZ-96 DNA Methylation-Gold kit (Zymo Research, Orange, CA, USA). The polymerase chain reaction (PCR) was performed in a 25 μl total volume. The final concentrations were: 0.05 U/μl for Qiagen HotStarTaq DNA Polymerase with 1.25 × PCR buffer, plus 1.0 mM of magnesium chloride, 0.2 μM for each primer and 0.50 mM for dNTP mix (Roche NucleoMix). PCR started with an initial denaturation of 15 minutes at 95°C followed by 45 cycles of 20 s at 95°C, 30 s at 56°C and 60 s at 72°C; the reaction finished with 5 minutes at 72°C. PCR products were purified and sequenced by pyrosequencing as previously described [33] using 0.3 μM sequencing primer. Primer sequences were previously published [34,35], and are the following: forward, 5′-TTT TGA GTT AGG TGT GGG ATA TA-3′; reverse, 5′-Biotin-AAA ATC AAA AAA TTC CCT TTC-3′; and sequencing, 5′-AGT TAG GTG TGG GAT ATA GT-3′. The targeted region comprised three CpGs located in the 5′ region of *LINE-1* elements [19] and had the following bisulfite-treated DNA sequence: 5′-TT**C**/**T**GTGGTG**C**/**T**GT**C**/**T**G-3′, where C/T corresponded to methylated (C) and unmethylated (T) cytosine at each CpG site. Since the %meth levels of the three CpG sites were highly correlated between each other (r = 0.57 - 0.83, *P* <0.0001), the mean %meth of combined *LINE-1* CpG sites was calculated for each subject and used in the association analyses.

### Statistical analysis

Non-normally distributed phenotypes were log_10_ or negative inverse transformed. The general linear model (GLM) and the type III sum of squares (all subjects: sex included in the model; sex-specific: unadjusted) were used to compare the mean phenotype levels between subjects without (MetS-) and with (MetS+) MetS. Multiple linear regression analyses were performed to predict each MetS-related phenotype and MetS *per se* while including *LINE-1*%meth as an independent variable along with MetS-related potential confounding factors (see the Results section for more details about adjustments). After dividing the subjects into quartiles based on their *LINE-1*%meth and dichotomized by MetS- and MetS + groups, a logistic regression analysis was performed to compute odds ratios (ORs) and Wald’s confidence limits to evaluate whether *LINE-1*%meth quartiles can predict MetS risk. Finally, a multiple linear regression analysis was used to predict *LINE-1*%meth levels where age, sex and smoking were included in the model as independent variables. The statistically significant *P* value was set at 0.05. Statistical analyses were performed using SAS software V.9.2 (SAS Institute, Cary, NC, USA).

## Results

### Characteristics of the study subjects in MetS- and MetS + groups

The characteristics of the subjects are presented in Table 1. No difference was observed in the mean age and smoking frequencies between MetS groups. Study subjects were severely obese with a mean body mass index of about 52 kg/m^2^ and a mean age of about 35 years. Women in the MetS + group had higher mean body mass index and waist circumference values compared to MetS- women. As expected, all the mean MetS-related phenotypes differed significantly between MetS groups.

**Table 1:** Subject characteristics according to metabolic syndrome (MetS) status

	**All subjects**		**Men**		**Premenopausal women**	
**Phenotypes**^**a**^	**MetS-**	**MetS+**	**MetS-**	**MetS+**	**MetS-**	**MetS+**
No. of subjects (*n*)	98	88	14	20	84	68
Smokers (*n*)	21 (21.4%)	22 (25.6%)	2 (14.3%)	4 (21.1%)	19 (22.6%)	18 (26.9%)
Age (years)	34.9 ± 8.1	35.3 ± 7.3	32.2 ± 8.9	36.8 ± 9.2	35.4 ± 7.9	34.9 ± 6.7
Body Mass Index (kg/m^2^)^b^	49.8 ± 8.4	53.8 ± 10.8^*^	54.5 ± 8.8	56.8 ± 13.1	49.0 ± 8.1	52.9 ± 9.9^**^
Waist circumference (cm)	130.1 ± 17.0	139.4 ± 19.6^**^	151.6 ± 15.6	160.7 ± 20.7	126.8 ± 14.7	132.9 ± 14.0^*^
Fasting glucose (mmol/l) ^b^	4.87 ± 0.39	5.68 ± 0.87^***^	5.06 ± 0.33	6.16 ± 0.75^***^	4.84 ± 0.39	5.53 ± 0.86^***^
Triglycerides (mmol/l) ^b^	1.13 ± 0.37	2.20 ± 1.14^***^	1.08 ± 0.31	2.42 ± 1.41^***^	1.13 ± 0.38	2.13 ± 1.06^***^
High-density lipoprotein-cholesterol (mmol/l)	1.47 ± 0.26	1.09 ± 0.20^***^	1.27 ± 0.17	0.94 ± 0.15^***^	1.51 ± 0.38	1.13 ± 0.20^***^
Systolic blood pressure (mmHg)	130.2 ± 13.4	144.6 ± 17.2^***^	132.1 ± 12.8	150.2 ± 19.4^**^	129.9 ± 13.5	143.0 ± 16.4^***^
Diastolic blood pressure (mmHg) ^b^	79.9 ± 9.3	90.4 ± 11.9^***^	78.8 ± 7.8	89.7 ± 13.2^*^	80.0 ± 8.7	90.6 ± 11.6^***^

^a^Data are shown as mean ± SD or *n*.

^b^Non-normally distributed phenotypes were transformed for the general linear model analysis: body mass index (negative inverse: -1/(X)), fasting glucose (-1/(X)), triglycerides (-1/(1 + X)) and diastolic blood pressure (-1/(X)).

^*^P <0.05, ^**^P <0.01, ^***^P <0.0001; for differences in mean phenotype levels between MetS- and MetS + groups.

### Relationship between *LINE-1*%meth levels, MetS and its related phenotypes

The mean *LINE-1*%meth in VAT of 186 severely obese subjects was of 75.8% (SD = 3.0%) with values ranging from 67.3 to 85.4%. Regression coefficients obtained specifically for *LINE-1*%meth in predictive models for each MetS-related phenotype and MetS *per se* are shown in Table 2. Negative associations were seen between *LINE-1*%meth levels and fasting glucose levels (β (95% confidence interval) = -0.04 (-0.08 to -0.01); *P* = 0.03) and DBP (-0.65 (-1.26 to -0.05); *P* = 0.03) when age, sex, waist circumference and smoking were included in the model. *LINE-1*%meth levels were also significantly predicting MetS *per se* (-0.04 (-0.06 to -0.01); *P* = 0.004) when adjusted for age, sex and smoking. Of note, CpG site specific complementary analyses revealed that methylation levels of the first CpG site investigated in this assay were more closely related to the previous MetS phenotypes (fasting glucose: β = -0.04, *P* = 0.007; DBP: β = -0.52, *P* = 0.04; and MetS *per se*: β = -0.04, *P* = 0.0002), and also with the SBP (β = -0.88, *P* = 0.01), as compared to methylation levels of the second and the third CpG sites (*P* >0.05 for the whole model; data not shown).

**Table 2:** **Multiple linear regression analysis predicting metabolic syndrome (MetS)-related phenotypes while including*LINE-1*methylation level (*LINE-1*%meth) in the predictive model**

**Dependent variables**	***LINE-1*%meth**		
	***N***	**β (95% CI)**^**model**^	***P*value**
Waist circumference (cm)	180	-0.51 (-1.33 to 0.32)^A^	0.23
Fasting glucose (mmol/l)	179	-0.04 (-0.08 to -0.01)^B^	0.03
Triglycerides (mmol/l)	179	0.01 (-0.04 to 0.06)^B^	0.64
High-density lipoprotein-cholesterol (mmol/l)	179	0.01 (-0.003 to 0.03)^B^	0.13
Systolic blood pressure (mmHg)	180	-0.71 (-1.55 to 0.13)^B^	0.10
Diastolic blood pressure (mmHg)	180	-0.65 (-1.26 to -0.05)^B^	0.03
MetS (*n*)	184	-0.04 (-0.06 to -0.01)^A^	0.004

Only coefficients (β), 95% confidence intervals (95% CI) and *P* values accounted for by the independent variable *LINE-1*%meth levels are shown in the table. The coefficient β corresponds to the change in the dependent variable per unit change in *LINE-1*%meth, given that all the other variables in the model remain constant.

Model A: *LINE-1*%meth, age, sex and smoking.

Model B: *LINE-1*%meth, age, sex, smoking and waist circumference.

### Prediction of MetS risk using quartiles of *LINE-1*%meth in VAT

The study subjects were divided into quartiles (Q) based on their *LINE-1*%meth levels (Q1: 67.3% to 73.8%; Q2: 73.8% to 75.0%; Q3: 75.0% to 77.3%; Q4: 77.3% to 85.4%) and dichotomized into MetS- and MetS + groups. ORs were computed to evaluate whether some *LINE-1*%meth quartiles may be associated with greater risk of MetS, while taking the higher %meth quartile as the reference group. Greater risks were observed in the first (OR (95% confidence limits (CL); Q1: OR = 4.37 (1.59 to 12.01), *P* = 0.004) and the second (Q2: OR = 4.76 (1.76 to 12.90), *P* = 0.002) quartiles compared to Q4 (1.00), which was not the case for the third quartile (Q3: OR = 1.82 (0.70 to 4.71), *P* = 0.22) when adjusting for age, sex and smoking (Table 3). While using a cut-off point of 75.0% for *LINE-1*%meth levels, the sensitivity and specificity of correctly categorizing subjects as having or not having MetS were 60.2% and 59.2%, respectively. Although the %meth cut-off point that would confer greater risk for MetS seems slightly different in additional site specific analyses, practically the same observation could be drawn, that is, quartiles with lower %meth levels for each CpG site were associated with greater MetS risk (Q1 for CpG_1,2_: OR = 2.82 to 8.34, *P* <0.04; Q2 for CpG_1,2_: OR = 2.80 to 8.16, *P* <0.04; Q3 for CpG_1_: OR = 2.71, *P* = 0.047), as compared to the highest %meth quartile (Q4) (data not shown).

**Table 3:** **Logistic regression analysis evaluating the association between*LINE-1*methylation level (*LINE-1*%meth) quartiles and metabolic syndrome (MetS) risk**

***LINE-1*%meth quartiles**	**MetS-,*n*** **= 98**	**MetS+,*n*** **= 86**	**Wald’s*χ***^**2**^ **= 12.3,*P*** **= 0.007**^**a**^, **OR (95% CL)**	***P*value**
Q1: 67.3% to 73.8%	21 (0.22)	25 (0.29)	4.37 (1.59 to 12.02)	0.004
Q2: 73.8% to 75.0%	19 (0.19)	27 (0.31)	4.76 (1.76 to 12.90)	0.002
Q3: 75.0% to 77.3%	28 (0.29)	18 (0.21)	1.82 (0.70 to 4.71)	0.22
Q4: 77.3% to 85.0%	30 (0.31)	16 (0.18)	1.00	-

Odds ratios and *P* values computed by the logistic regression analysis were obtained while including potential confounding factors in the model, namely age, sex and smoking. The fourth *LINE-1*%meth quartile (Q4) was set as the reference group.

^a^Corresponds to the Wald’s *χ*^2^ and *P* value for the effect of *LINE-1*%meth quartiles. For the whole model, Wald’s *χ*^2^ = 14.0 and *P* value = 0.03.

*CL* = Wald’s confidence limits.

### Predictors of *LINE-1*%meth levels in VAT

The contribution of potential confounding factors in *LINE-1*%meth variation was tested using a multiple linear regression model including age, sex and smoking. The variance was partly accounted for by sex (β (95% confidence interval) = -3.21 (-4.27 to -2.15); *P* <0.0001; lower in women). Other factors were not significant (age: 0.04 (-0.02 to 0.09), *P* = 0.17; smoking: 0.33 (-0.64 to 1.29), *P* = 0.50).

## Discussion

This study revealed that *LINE-1*%meth levels in VAT were associated negatively with fasting plasma glucose, blood pressure and MetS *per se* using the IDF definition [30]. Subjects situated in the quartiles with lower *LINE-1*%meth levels had significantly greater risk to be affected by MetS when adjusted for age, sex and smoking. Sex was a significant predictor of *LINE-1*%meth levels in VAT of the study subjects, which was not the case for age and smoking. To the best of our knowledge, this is the first study analyzing *LINE-1*%meth levels in adipose tissue. Most of the published studies used peripheral blood cells [18,23,25,36-38], tumorous vs non-tumorous tissues [39,40], and placental tissues [41] for *LINE-1* methylation analysis. *LINE-1*%meth levels observed in VAT of the study subjects were comparable to levels reported in previous epidemiological studies using white blood cells (WBC) and the same methylation quantification method [18,23]. It thus suggests that *LINE-1*%meth levels between VAT and WBC are comparable, even though the correlation between both compartments was not tested in the present study. Although further studies would be needed to investigate whether *LINE-1*%meth levels in WBC are associated with MetS risk, as a more convenient biological sample for clinical and epidemiological purposes, this study underlines the presence of DNA methylation heterogeneity in the VAT of severe obese individuals. This finding supports the hypothesis of potential epigenetic changes in VAT, which may contribute to the development of metabolic perturbations in the presence of abdominal obesity. Since differential gene expression patterns were previously observed in VAT of obese with versus without MetS [7], gene-specific DNA methylation analysis in VAT are warranted to better understand the contribution of epigenetics in VAT function and, consequently, in obesity-related metabolic disorders.

The study results showed higher plasma glucose and DBP levels, along with greater risk for MetS, in presence of lower *LINE-1*%meth levels in VAT. However, this relationship is in the opposite direction of what Pearce *et al.* reported [24]. In their samples of 228 Caucasian individuals aged 49 to 51 years from the Newcastle Thousand Families Study (Newcastle upon Tyne, UK), they found a positive association between peripheral blood *LINE-1*%meth and fasting glucose levels. They also observed positive and negative associations, respectively, with plasma triglyceride and HDL-cholesterol levels after adjustment for sex [24]. In contrast, Kim *et al.* reported no significant association between blood *LINE-1*%meth levels and fasting glucose, triglyceride, HDL-cholesterol, SBP and DBP in healthy male and female Koreans [42]. Since their study sample was relatively small (*n* = 86) and that no adjustments for sex were performed, it is difficult to clearly delineate the direction of the relationship between *LINE-1*%meth and MetS-related phenotypes. However, since most studies reported that lower *LINE-1*%meth levels in peripheral blood cells were associated with lower HDL-cholesterol levels [25] and with higher risk of incident ischemic heart disease and stroke [23], it is tempting to suggest that lower *LINE-1*%meth levels may be associated with MetS and its related phenotypes.

Regarding potential predictors of *LINE-1*%meth, lower methylation levels were observed in VAT of women as compared to men in this study, which was also reported in several studies using WBC for *LINE-1*%meth quantification [19,38,43,44]. The reason for this sex difference still remains to be clarified, but it does not appear to be related to male/female hormone differences [44,45]. While there is growing evidence that WBC global DNA methylation reduces with aging, several studies did not observe an association between *LINE-1*%meth levels with either age [38,44], or active cigarette smoking in adults [38]. Consistent with these previous studies, age and smoking were not significant predictors of *LINE-1*%meth levels in VAT of the present study subjects.

As reported in a previous study, many genes were differentially expressed in VAT of MetS- versus MetS + severely obese men [7] and epigenetic mechanisms have been postulated as potentially mediating differential expressions [33]. There is recent evidence supporting a correlation between *LINE-1* and gene-specific %meth levels, which would depend upon the genomic region and type of tissue analyzed [46]. However, the nature of this relationship still remains underexplored. The relevance of *LINE-1* methylation quantification as a marker of global DNA methylation is supported by the fact that *LINE-1* is the most prevalent repetitive sequence in the human genome (approximately 17% of the genome) [20] and that about one-third of DNA methylation in the genome occurs in these elements [35]. Some reports have also shown that *LINE-1*%meth levels significantly correlated with other methods assessing global DNA methylation, such as genomic 5-methyl cytosine content [47] and luminometric methylation assay (LUMA) [46]. Bisulfite sequencing of repetitive elements, as performed here for *LINE-1* elements, is a quicker and easier method to assess global DNA methylation as compared to genomic 5-methyl cytosine quantification [35]. Thus, all the above observations would support the utility of assessing *LINE-1*%meth as a marker of global DNA methylation in VAT of our study subjects, and suggest that the variability observed in *LINE-1*%meth levels may be suggestive of concomitant variability in gene-specific %meth levels potentially associated with differential gene expression and MetS phenotypes. It should be mentioned that methylation quantification of other repetitive elements, such as Alu repeats, is sometimes performed to assess global DNA methylation [18], which was not measured in this study. Correlations between *LINE-1* and Alu methylation levels were observed in some but not all studies [18,48] and may depend on the type of tissues examined and the responses to cellular stressors and environmental exposures [18]. *LINE-1* and Alu repeats represent distinct measures of dispersed DNA methylation [19] and it is unknown whether the same associations observed in this study would be seen with Alu methylation levels. *LINE-1* methylation assessment was chosen in this study over Alu because of previous associations between *LINE-1* methylation levels and MetS phenotypes [24,25], as well as with ischemic heart disease and stroke [23]. Another reason for investigating *LINE-1* elements is its function. There are approximately 3,000 to 5,000 full length (approximately 6 kb) *LINE-1* sequences throughout the genome, which comprise the 5′ region targeted in the *LINE-1*%meth analysis, in which approximately 60 to 100 elements are still capable of retrotransposition [49]. It has been postulated in cancer that demethylation of *LINE-1* elements may increase their retrotransposable activity, induce genomic instability, and deregulate transcriptional activity of specific genes [50]. Whether such a portrait would also be seen in other common complex diseases is unknown.

Some study limitations need to be outlined, such as the possibility of reverse causality [17]. It is indeed possible that the development of obesity-related metabolic complications may have induced changes in *LINE-1*%meth rather than the reverse. Furthermore, DNA methylation of *LINE-1* elements has been associated with several environmental exposures, such as air pollution, metal exposures, persistent organic pollutants, as well as alcohol and dietary folate consumption (reviewed in [38]). Whether these confounding factors have influenced *LINE-1*%meth variability in this study and consequently changed the relationship seen with MetS is unknown. It may however need some concerns since several persistent organic pollutants may accumulate in adipose tissue, which have been attributed with obesity development, type 2 diabetes and related metabolic impairments in human and animal models [51,52]. Also, VAT is a heterogeneous tissue composed of different cell types, including infiltrated lymphocytes [53], which are increased in presence of obesity and associated with insulin resistance [54]. It has been shown that the percentage of lymphocytes in blood cell counts was negatively associated with blood *LINE-1*%meth levels [18]. Whether our MetS + subjects have greater lymphocyte infiltration in their VAT, which may influence *LINE-1*%meth values obtained in this study, is a possibility which warrants further investigation. Finally, Zhang *et al.* have previously reported that some variability in *LINE-1* methylation levels exist in peripheral leukocytes among cancer-free individuals of different ethnicities [43]. These authors proposed that genetic polymorphisms in the folate metabolism, which provides methyl groups for DNA methylation [55], or other unknown genetic and environmental factors, may contribute to ethnic differences in global DNA methylation [43]. It thus suggests that associations observed among Caucasians in the present study may not necessarily be generalized to all ethnic groups and may potentially be influenced by genetic polymorphisms.

## Conclusions

In summary, this study revealed that lower *LINE-1*%meth levels in VAT of non-diabetic severely obese subjects was associated with higher plasma glucose and DBP levels, and with a greater risk to be affected by MetS. This investigation also point out to the necessity to pursue gene-specific methylation analysis among differentially expressed genes in the VAT of severely obese subjects MetS- and MetS + [7] to better understand the involvement of epigenetic regulation in the development of obesity-related metabolic complications.

## Competing interests

The authors declare that they have no competing interests.

## Authors’ contributions

VT initiated the study, performed the statistical analyses, interpreted the data and drafted the manuscript. AT, YD, and LP participated to the elaboration of the study design. AB was in charge of the methylation analysis. SM, SB, OL, LB sampled blood and adipose tissue from the study subjects. MCV conceived and designed the study. All authors read and approved the final manuscript.

## References

[B1] Alberti KG Eckel RH Grundy SM Zimmet PZ Cleeman JI Donato KA Fruchart JC James WP Loria CM Smith SC Harmonizing the metabolic syndrome: a joint interim statement of the International Diabetes Federation Task Force on Epidemiology and Prevention; National Heart, Lung, and Blood Institute; American Heart Association; World Heart Federation; International Atherosclerosis Society; and International Association for the Study of Obesity Circulation 2009 120 1640 1645 19805654 10.1161/CIRCULATIONAHA.109.192644

[B2] Mathieu P Lemieux I Despres JP Obesity, inflammation, and cardiovascular risk Clin Pharmacol Ther 2010 87 407 416 20200516 10.1038/clpt.2009.311

[B3] Pajunen P Kotronen A Korpi-Hyovalti E Keinanen-Kiukaanniemi S Oksa H Niskanen L Saaristo T Saltevo JT Sundvall J Vanhala M Uusitupa M Peltonen M Metabolically healthy and unhealthy obesity phenotypes in the general population: the FIN-D2D Survey BMC Publ Health 2011 11 754 10.1186/1471-2458-11-754 PMC3198943 21962038

[B4] Primeau V Coderre L Karelis AD Brochu M Lavoie ME Messier V Sladek R Rabasa-Lhoret R Characterizing the profile of obese patients who are metabolically healthy Int J Obes (Lond) 2011 35 971 981 20975726 10.1038/ijo.2010.216

[B5] Bluher M Adipose tissue dysfunction in obesity Exp Clin Endocrinol Diabetes 2009 117 241 250 19358089 10.1055/s-0029-1192044

[B6] Bluher M The distinction of metabolically 'healthy' from 'unhealthy' obese individuals Curr Opin Lipidol 2010 21 38 43 19915462 10.1097/MOL.0b013e3283346ccc

[B7] Bouchard L Tchernof A Deshaies Y Marceau S Lescelleur O Biron S Vohl MC ZFP36: a promising candidate gene for obesity-related metabolic complications identified by converging genomics Obes Surg 2007 17 372 382 17546847 10.1007/s11695-007-9067-5

[B8] Hardy OT Perugini RA Nicoloro SM Gallagher-Dorval K Puri V Straubhaar J Czech MP Body mass index-independent inflammation in omental adipose tissue associated with insulin resistance in morbid obesity Surg Obes Relat Dis 2011 7 60 67 20678967 10.1016/j.soard.2010.05.013 PMC2980798

[B9] Klimcakova E Roussel B Kovacova Z Kovacikova M Siklova-Vitkova M Combes M Hejnova J Decaunes P Maoret JJ Vedral T Viguerie N Bourlier V Bouloumie A Stich V Langin D Macrophage gene expression is related to obesity and the metabolic syndrome in human subcutaneous fat as well as in visceral fat Diabetologia 2011 54 876 887 21267541 10.1007/s00125-010-2014-3

[B10] Delisle H Programming of chronic disease by impaired fetal nutrition: evidence and implications for policy and intervention strategies. Report of the Department of Nutrition for Health and Development, Department of Noncommunicable Disease Prevention and Health Promotion 2002 World Health Organization, Geneva

[B11] McMillen IC Robinson JS Developmental origins of the metabolic syndrome: prediction, plasticity, and programming Physiol Rev 2005 85 571 633 15788706 10.1152/physrev.00053.2003

[B12] Symonds ME Sebert SP Hyatt MA Budge H Nutritional programming of the metabolic syndrome Nat Rev Endocrinol 2009 5 604 610 19786987 10.1038/nrendo.2009.195

[B13] Fall CH Evidence for the intra-uterine programming of adiposity in later life Ann Hum Biol 2011 38 410 428 21682572 10.3109/03014460.2011.592513 PMC3428869

[B14] Muhlhausler B Smith SR Early-life origins of metabolic dysfunction: role of the adipocyte Trends Endocrinol Metab 2009 20 51 57 19095460 10.1016/j.tem.2008.10.006

[B15] Bruce KD Cagampang FR Epigenetic priming of the metabolic syndrome Toxicol Mech Methods 2011 21 353 361 21495873 10.3109/15376516.2011.559370

[B16] Campion J Milagro FI Martinez JA Individuality and epigenetics in obesity Obes Rev 2009 10 383 392 19413700 10.1111/j.1467-789X.2009.00595.x

[B17] Relton CL Davey SG Epigenetic epidemiology of common complex disease: prospects for prediction, prevention, and treatment PLoS Med 2010 7 e1000356 21048988 10.1371/journal.pmed.1000356 PMC2964338

[B18] Zhu ZZ Hou L Bollati V Tarantini L Marinelli B Cantone L Yang AS Vokonas P Lissowska J Fustinoni S Pesatori AC Bonzini M Apostoli P Costa G Bertazzi PA Chow WH Schwartz J Baccarelli A Predictors of global methylation levels in blood DNA of healthy subjects: a combined analysis Int J Epidemiol 2010 41 126 139 20846947 10.1093/ije/dyq154 PMC3304518

[B19] Nelson HH Marsit CJ Kelsey KT Global methylation in exposure biology and translational medical science Environ Health Perspect 2011 119 1528 1533 21669556 10.1289/ehp.1103423 PMC3226501

[B20] Lander ES Linton LM Birren B Nusbaum C Zody MC Baldwin J Devon K Dewar K Doyle M FitzHugh W Funke R Gage D Harris K Heaford A Howland J Kann L Lehoczky J LeVine R McEwan P McKernan K Meldrim J Mesirov JP Miranda C Morris W Naylor J Raymond C Rosetti M Santos R Sheridan A Sougnez C Initial sequencing and analysis of the human genome Nature 2001 409 860 921 11237011 10.1038/35057062

[B21] Sunami E de MM Vu A Turner RR Hoon DS LINE-1 hypomethylation during primary colon cancer progression PLoS One 2011 6 e18884 21533144 10.1371/journal.pone.0018884 PMC3077413

[B22] Zhu ZZ Sparrow D Hou L Tarantini L Bollati V Litonjua AA Zanobetti A Vokonas P Wright RO Baccarelli A Schwartz J Repetitive element hypomethylation in blood leukocyte DNA and cancer incidence, prevalence, and mortality in elderly individuals: the Normative Aging Study Cancer Causes Control 2011 22 437 447 21188491 10.1007/s10552-010-9715-2 PMC3752839

[B23] Baccarelli A Wright R Bollati V Litonjua A Zanobetti A Tarantini L Sparrow D Vokonas P Schwartz J Ischemic heart disease and stroke in relation to blood DNA methylation Epidemiology 2010 21 819 828 20805753 10.1097/EDE.0b013e3181f20457 PMC3690659

[B24] Pearce MS McConnell JC Potter C Barrett LM Parker L Mathers JC Relton CL Global LINE-1 DNA methylation is associated with blood glycaemic and lipid profiles Int J Epidemiol 2012 41 210 217 22422454 10.1093/ije/dys020 PMC3304536

[B25] Cash HL McGarvey ST Houseman EA Marsit CJ Hawley NL Lambert-Messerlian GM Viali S Tuitele J Kelsey KT Cardiovascular disease risk factors and DNA methylation at the LINE-1 repeat region in peripheral blood from Samoan Islanders Epigenetics 2011 6 1257 1264 21937883 10.4161/epi.6.10.17728 PMC3225843

[B26] Marceau P Hould FS Simard S Lebel S Bourque RA Potvin M Biron S Biliopancreatic diversion with duodenal switch World J Surg 1998 22 947 954 9717420 10.1007/s002689900498

[B27] Report of the expert committee on the diagnosis and classification of diabetes mellitus Diabetes Care 2003 26 Suppl 1 S5 S20 12502614 10.2337/diacare.26.2007.s5

[B28] Vohl MC Houde A Lebel S Hould FS Marceau P Effects of the peroxisome proliferator-activated receptor-gamma co-activator-1 Gly482Ser variant on features of the metabolic syndrome Mol Genet Metab 2005 86 300 306 16122961 10.1016/j.ymgme.2005.07.002

[B29] Vohl MC Sladek R Robitaille J Gurd S Marceau P Richard D Hudson TJ Tchernof A A survey of genes differentially expressed in subcutaneous and visceral adipose tissue in men Obes Res 2004 12 1217 1222 15340102 10.1038/oby.2004.153

[B30] Alberti KG Zimmet P Shaw J Metabolic syndrome--a new world-wide definition. A Consensus Statement from the International Diabetes Federation Diabet Med 2006 23 469 480 16681555 10.1111/j.1464-5491.2006.01858.x

[B31] Executive Summary of The Third Report of The National Cholesterol Education Program (NCEP) Expert Panel on Detection, Evaluation, And Treatment of High Blood Cholesterol In Adults (Adult Treatment Panel III) JAMA 2001 285 2486 2497 11368702 10.1001/jama.285.19.2486

[B32] England R Pettersson M Pyro Q-CpG: quantitative analysis of methylation in multiple CpG sites by pyrosequencing Nature Methods 2005 2 10.1038

[B33] Turcot V Bouchard L Faucher G Tchernof A Deshaies Y Perusse L Belisle A Marceau S Biron S Lescelleur O Biertho L Vohl MC DPP4 gene DNA methylation in the omentum is associated with its gene expression and plasma lipid profile in severe obesity Obesity (Silver Spring) 2011 19 388 395 20847730 10.1038/oby.2010.198

[B34] Kile ML Baccarelli A Tarantini L Hoffman E Wright RO Christiani DC Correlation of global and gene-specific DNA methylation in maternal-infant pairs PLoS One 2010 5 e13730 21060777 10.1371/journal.pone.0013730 PMC2966409

[B35] Yang AS Estecio MR Doshi K Kondo Y Tajara EH Issa JP A simple method for estimating global DNA methylation using bisulfite PCR of repetitive DNA elements Nucleic Acids Res 2004 32 e38 14973332 10.1093/nar/gnh032 PMC373427

[B36] Baccarelli A Tarantini L Wright RO Bollati V Litonjua AA Zanobetti A Sparrow D Vokonas P Schwartz J Repetitive element DNA methylation and circulating endothelial and inflammation markers in the VA normative aging study Epigenetics 2010 5 222 228 10.4161/epi.5.3.11377 PMC3155741 20305373

[B37] Choi JY James SR Link PA McCann SE Hong CC Davis W Nesline MK Ambrosone CB Karpf AR Association between global DNA hypomethylation in leukocytes and risk of breast cancer Carcinogenesis 2009 30 1889 1897 19584139 10.1093/carcin/bgp143 PMC2783000

[B38] Terry MB Delgado-Cruzata L Vin-Raviv N Wu HC Santella RM DNA methylation in white blood cells: association with risk factors in epidemiologic studies Epigenetics 2011 6 828 837 21636973 10.4161/epi.6.7.16500 PMC3154425

[B39] Irahara N Nosho K Baba Y Shima K Lindeman NI Hazra A Schernhammer ES Hunter DJ Fuchs CS Ogino S Precision of pyrosequencing assay to measure LINE-1 methylation in colon cancer, normal colonic mucosa, and peripheral blood cells J Mol Diagn 2010 12 177 183 20093385 10.2353/jmoldx.2010.090106 PMC2871724

[B40] Pavicic W Joensuu EI Nieminen T Peltomaki P LINE-1 hypomethylation in familial and sporadic cancer J Mol Med (Berl) 2012 10.1007/s00109-011-0854-z PMC3383956 22228215

[B41] Wilhelm-Benartzi CS Houseman EA Maccani MA Poage GM Koestler DC Langevin SM Gagne LA Banister C Padbury JF Marsit CJ In utero exposures, infant growth, and DNA methylation of repetitive element and developmentally related genes in human placenta Environ Health Perspect 2012 120 296 302 22005006 10.1289/ehp.1103927 PMC3279448

[B42] Kim KY Kim DS Lee SK Lee IK Kang JH Chang YS Jacobs DR Steffes M Lee DH Association of low-dose exposure to persistent organic pollutants with global DNA hypomethylation in healthy Koreans Environ Health Perspect 2010 118 370 374 20064773 10.1289/ehp.0901131 PMC2854765

[B43] Zhang FF Cardarelli R Carroll J Fulda KG Kaur M Gonzalez K Vishwanatha JK Santella RM Morabia A Significant differences in global genomic DNA methylation by gender and race/ethnicity in peripheral blood Epigenetics 2011 6 623 629 21739720 10.4161/epi.6.5.15335 PMC3230547

[B44] El-Maarri O Walier M Behne F van UJ Singer H Diaz-Lacava A Nusgen N Niemann B Watzka M Reinsberg J van dV Wienker T Stoffel-Wagner B Schwaab R Oldenburg J Methylation at global LINE-1 repeats in human blood are affected by gender but not by age or natural hormone cycles PLoS One 2011 6 e16252 21311577 10.1371/journal.pone.0016252 PMC3023801

[B45] Singer H Walier M Nusgen N Meesters C Schreiner F Woelfle J Fimmers R Wienker T Kalscheuer VM Becker T Schwaab R Oldenburg J El-Maarri O Methylation of L1Hs promoters is lower on the inactive X, has a tendency of being higher on autosomes in smaller genomes and shows inter-individual variability at some loci Hum Mol Genet 2012 21 219 235 21972244 10.1093/hmg/ddr456 PMC3235015

[B46] Poage GM Houseman EA Christensen BC Butler RA Avissar-Whiting M McClean MD Waterboer T Pawlita M Marsit CJ Kelsey KT Global hypomethylation identifies loci targeted for hypermethylation in head and neck cancer Clin Cancer Res 2011 17 3579 3589 21505061 10.1158/1078-0432.CCR-11-0044 PMC3107873

[B47] Weisenberger DJ Campan M Long TI Kim M Woods C Fiala E Ehrlich M Laird PW Analysis of repetitive element DNA methylation by MethyLight Nucleic Acids Res 2005 33 6823 6836 16326863 10.1093/nar/gki987 PMC1301596

[B48] Wu HC Delgado-Cruzata L Flom JD Kappil M Ferris JS Liao Y Santella RM Terry MB Global methylation profiles in DNA from different blood cell types Epigenetics 2011 6 76 85 20890131 10.4161/epi.6.1.13391 PMC3052916

[B49] Kazazian HH Goodier JL LINE drive. retrotransposition and genome instability Cell 2002 110 277 280 12176313 10.1016/s0092-8674(02)00868-1

[B50] Daskalos A Nikolaidis G Xinarianos G Savvari P Cassidy A Zakopoulou R Kotsinas A Gorgoulis V Field JK Liloglou T Hypomethylation of retrotransposable elements correlates with genomic instability in non-small cell lung cancer Int J Cancer 2009 124 81 87 18823011 10.1002/ijc.23849

[B51] Lee DH Persistent organic pollutants and obesity-related metabolic dysfunction: focusing on type 2 diabetes Epidemiol Health 2012 34 e2012002 22323980 10.4178/epih/e2012002 PMC3272548

[B52] Ruzzin J Petersen R Meugnier E Madsen L Lock EJ Lillefosse H Ma T Pesenti S Sonne SB Marstrand TT Malde MK Du ZY Chavey C Fajas L Lundebye AK Brand CL Vidal H Kristiansen K Froyland L Persistent organic pollutant exposure leads to insulin resistance syndrome Environ Health Perspect 2010 118 465 471 20064776 10.1289/ehp.0901321 PMC2854721

[B53] Sell H Eckel J Adipose tissue inflammation: novel insight into the role of macrophages and lymphocytes Curr Opin Clin Nutr Metab Care 2010 13 366 370 20473150 10.1097/MCO.0b013e32833aab7f

[B54] Winer DA Winer S Shen L Wadia PP Yantha J Paltser G Tsui H Wu P Davidson MG Alonso MN Leong HX Glassford A Caimol M Kenkel JA Tedder TF McLaughlin T Miklos DB Dosch HM Engleman EG B cells promote insulin resistance through modulation of T cells and production of pathogenic IgG antibodies Nat Med 2011 17 610 617 21499269 10.1038/nm.2353 PMC3270885

[B55] Ulrich CM Reed MC Nijhout HF Modeling folate, one-carbon metabolism, and DNA methylation Nutr Rev 2008 66 Suppl 1 S27 S30 18673484 10.1111/j.1753-4887.2008.00062.x

